# Delayed cytokinesis generates multinuclearity and potential advantages in the amoeba *Acanthamoebacastellanii* Neff strain

**DOI:** 10.1038/s41598-020-68694-9

**Published:** 2020-07-21

**Authors:** Théo Quinet, Ascel Samba-Louaka, Yann Héchard, Karine Van Doninck, Charles Van der Henst

**Affiliations:** 1 Laboratory of Evolutionary Genetics and Ecology, URBE, University of Namur, Namur, Belgium; 2 Laboratoire Ecologie et Biologie des Interactions, Equipe Microbiologie de L’Eau, Université de Poitiers, UMR CNRS 7267, 86073 Poitiers, France; 3 Research Unit in the Biology of Microorganisms (URBM), NARILIS, University of Namur (UNamur), Namur, Belgium; 4 Microbial Resistance and Drug Discovery, Center for Structural Biology (CSB), Flanders Institute for Biotechnology (VIB), Vrije Universiteit Brussel (VUB), Pleinlaan 2, Building E-3, 1050 Brussels, Belgium; 5 Structural Biology Brussels, Vrije Universiteit Brussel (VUB), Brussels, Belgium

**Keywords:** Parasitology, Evolutionary ecology, Microbial ecology

## Abstract

Multinuclearity is a widespread phenomenon across the living world, yet how it is achieved, and the potential related advantages, are not systematically understood. In this study, we investigate multinuclearity in amoebae. We observe that non-adherent amoebae are giant multinucleate cells compared to adherent ones. The cells solve their multinuclearity by a stretchy cytokinesis process with cytosolic bridge formation when adherence resumes. After initial adhesion to a new substrate, the progeny of the multinucleate cells is more numerous than the sibling cells generated from uninucleate amoebae. Hence, multinucleate amoebae show an advantage for population growth when the number of cells is quantified over time. Multiple nuclei per cell are observed in different amoeba species, and the lack of adhesion induces multinuclearity in diverse protists such as *Acanthamoeba* *castellanii*, *Vermamoeba* *vermiformis*, *Naegleria* *gruberi* and *Hartmannella* *rhysodes*. In this study, we observe that agitation induces a cytokinesis delay, which promotes multinuclearity. Hence, we propose the hypothesis that multinuclearity represents a physiological adaptation under non-adherent conditions that can lead to biologically relevant advantages.

## Introduction

The canonical view of eukaryotic cells is usually illustrated by an uninucleate organization. However, in the living world, cells harbouring multiple nuclei are common. This multinuclearity can have different origins, being either generated (i) by fusion events between uninucleate cells or by (ii) uninucleate cells that replicate their DNA content without cytokinesis. Animals, fungi, plants and protozoans possess such cells harbouring multiple nuclei, some of them being associated to a functional advantage.

Within the Amoebozoa, multinuclearity is found in many species, and can be an integral part of their life cycle. The infection cycle of the pathogenic amoeba *Entamoeba* *histolytica* involves tetranucleated cells^1^. In addition, within the giant amoeba *Pelomyxa* *carolinensis*, nuclei are “very abundant”^2^, containing from two to thousands of nuclei per cell^3^. Multinuclearity was also reported in two other amoebae: *Naegleria* *gruberi*^4^ and *Hartmannella* *rhysodes*^5^.

While multinuclearity appears common among Amoebozoa, there is a lack of evidence concerning its consequences and/or potentially associated function(s). Species within Amoebozoa therefore represent interesting model systems to study multinucleate cells. Hence, in this study, we investigate the generation of multinuclearity in an amoeba strain, the associated consequences and propose potential advantages. We use as cellular model the amoeba *Acanthamoeba* *castellanii*^6^ because its multinuclearity can be easily induced through agitation^7^. Moreover, *A.* *castellanii* is ubiquitous, being frequently found in aquatic environments such as sea water, swimming pools, cooling towers, tap waters or contact lens products^6^. *A.* *castellanii* is described as an opportunistic pathogen for human, inducing keratitis^8^, a corneal infection, when using contaminated contact lenses^9^. Despite the facts that *A.* *castellanii* represents a broadly used cellular infection model and a relevant human pathogen, improving the knowledge of its biology remains essential.

*Acanthamoeba* *castellanii* is characterized by a biphasic life cycle. Adherent trophozoites are motile, active in the uptake of size-compatible food particles, and are generally described as dividing by mitosis^10^. Upon harsh conditions such as starvation, desiccation, osmotic pressure variation or the presence of toxic compounds, the trophozoite differentiates into a cyst form^11^. This encystation process is mainly characterized by pseudopodia retraction, cellular detachment, dehydration of the cell body and the synthesis of a thick wall composed mainly of cellulose, protecting the amoeba from its environment^12^. To our knowledge, and in contrary of *E.* *histolytica*, no cysts of *A.* *castellanii* are described as tetranucleated^11^. However, previous studies showed that there was an increase in the proportion of multinucleate amoebae when *A.* *castellanii* cultures are under agitation^7^.

Knowing that several Amoebozoan species do contain multiple nuclei per single cell, we investigated the direct consequences of multinuclearity on population growth in *A.* *castellanii* by enumerating the number of cells over time. We hypothesized that it could represent a mechanism providing a specific advantage to the species during colonization.

## Results

### Absence of adhesion delays cytokinesis

**Figure 1:**
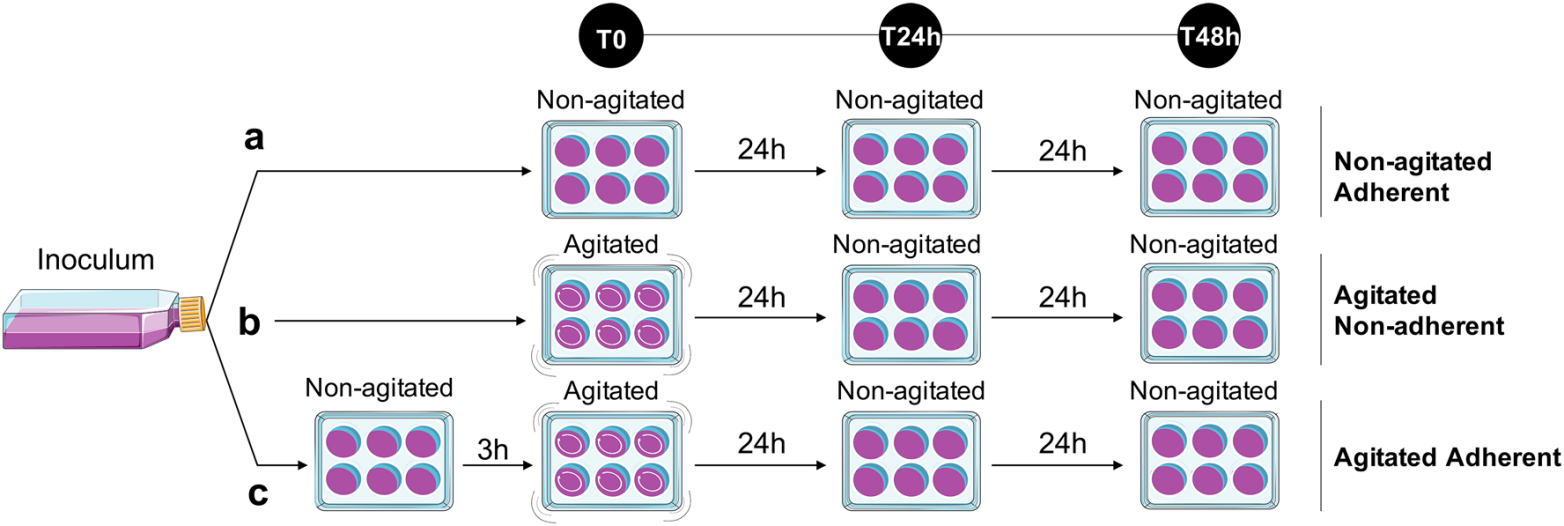
Experimental workflow to induce multinuclearity. The three conditions start from the same amoeba inoculum. (**a**) Non-agitated Adherent conditions. Amoebae are incubated without agitation throughout the experiment. (**b**) Agitated Non-adherent conditions. Amoebae are first incubated with agitation for 24 h and then without agitation for 24 h. (**c**) Agitated Adherent conditions. Amoeba cells are allowed to adhere for 3 h prior to be incubated with agitation for 24 h and then without agitation for 24 h.

Multinuclearity has been previously shown to be induced in *A.* *castellanii* by agitation^7^. In our study, amoebae in suspension (non-adherent) put under agitation are called “agitated non-adherent” cells (Fig. 1). The control of uninucleate cells are amoebae put under “non-agitated adherent” conditions. In order to confirm that the absence of adhesion per se, rather than the agitation of the medium itself, is the main factor inducing multinuclearity in our tested conditions, a second control used “agitated adherent” cells, for which amoebae are allowed to adhere on a substrate prior to agitation (Fig. 1).

**Figure 2:**
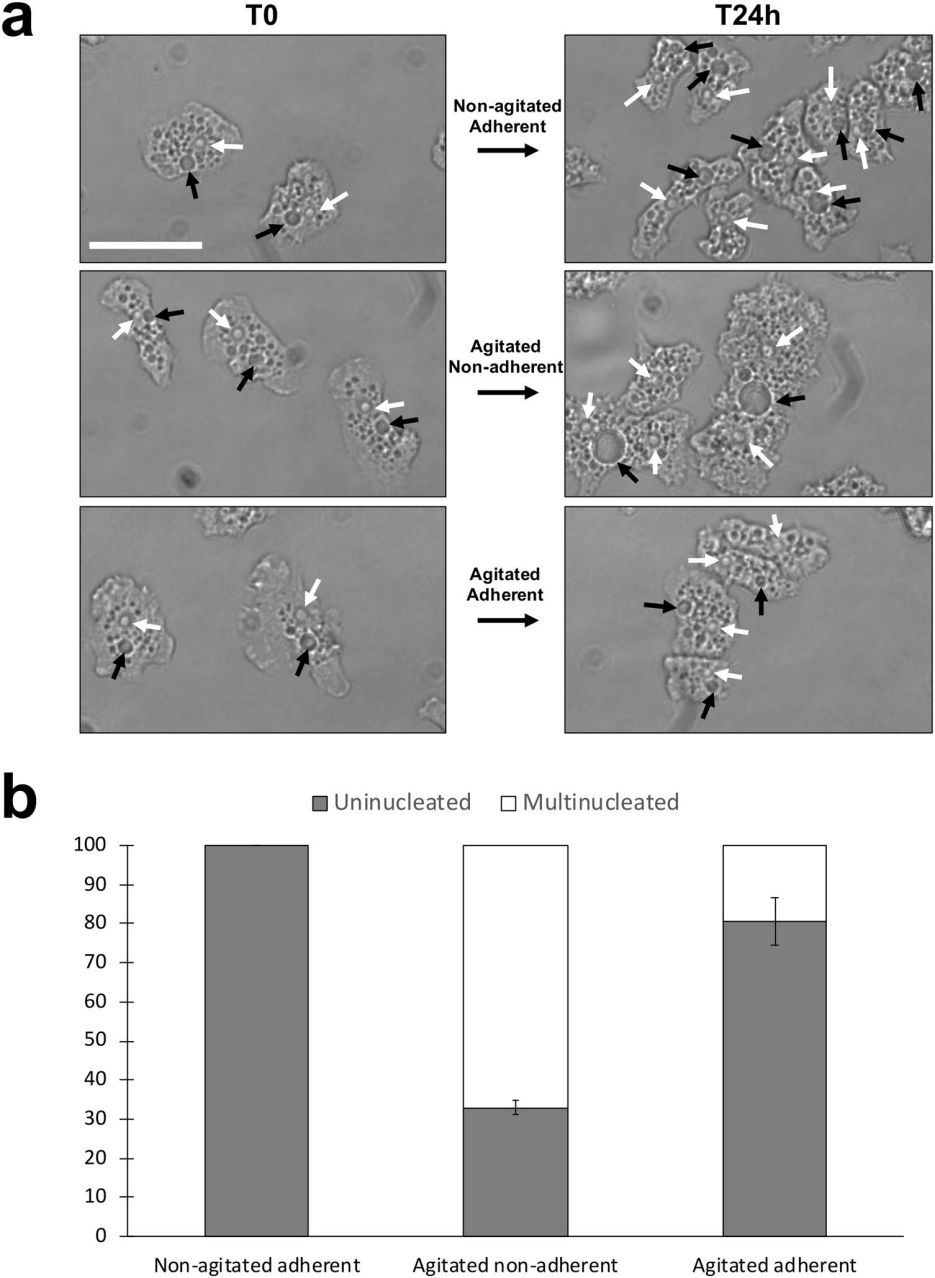
Lack of adhesion generates giant and multinucleate amoebae. (**a**) Adherent amoebae are cultivated without agitation for 24 h (upper panels); non-adherent amoebae are cultivated with agitation for 24 h (middle panels) and adherent amoebae are agitated for 24 h (lower panels). White arrows show the nuclei and black arrow depict the contractile vacuoles. Scale bar: 50 µm. (**b**) Quantification of the uninucleate (grey) and multinucleate (white) amoebae. The data are generated from three biological independent experiments. Error bars ± SEM.

Common morphologies of uninucleate *A.* *castellanii* amoebae were observed at the start of the experiment (“T0”) (Fig. 2a). After 24 h without agitation (Fig. 2a upper panel), *A.* *castellanii* divided and were therefore more numerous per field of view with similar cellular organization and morphology compared to the T0 (quantification is given in Fig. 2b). However, the ultrastructure and the morphology of non-adherent amoebae after 24 h under agitation were different (Fig. 2A middle panel). Non-adherent *A.* *castellanii* were giant and multinucleate (67.0% ± 1.9% of multinuclearity) when compared to standing adherent uninucleate amoebae for which no multinucleate cells are observed (number of amoebae counted = 1511) (Fig. 2b). While uninucleate amoebae measured on average 556.9 µm^2^ (± 68.9 µm^2^), multinucleate cells were larger (2,582.2 µm^2^ ± 581.1 µm^2^). The absolute size of the contractile vacuole was also larger (173.4 µm^2^ ± 75.1 µm^2^) than the one present in the uninucleate amoebae (25.0 µm^2^ ± 6.18 µm^2^). However, normalized to the size of the amoebae, contractile vacuoles had a comparable average size (22.3 µm^2^ ± 1.2 µm^2^ for uninucleate and 14.9 µm^2^ ± 3.71 µm^2^ for multinucleate amoebae) suggesting that giant amoebae were not dramatically impaired in their osmo-regulation capacity^13^. In the control conditions where amoebae were agitated but still adherent (Fig. 2a lower panel), the morphology of the amoebae were in the majority comparable to the non-agitated adherent conditions. However, for these agitated adherent *A.* *castellanii* amoebae, the quantification of uni-and multinucleate cells indicates that a minority of the population was multinucleated (Fig. 2b). The agitation could have detached some amoebae from their solid substrate.

**Figure 3:**
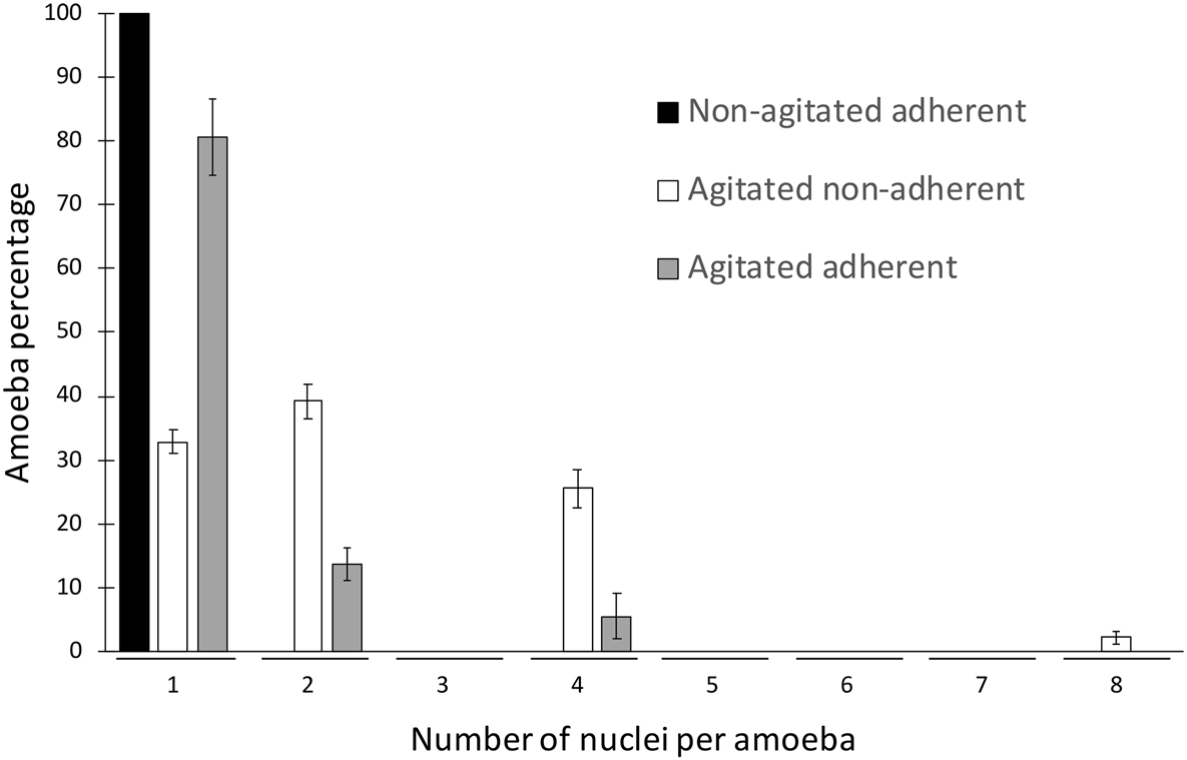
Frequency distribution of amoebae according to the number of nuclei. Non-agitated adherent (black), agitated non-adherent (white) and agitated adherent cells (grey). Amoebae were incubated in the related conditions for 24 h prior to direct observation under a microscope and subsequent quantification. Error bars ± SEM. Data are generated from three biological independent experiments, each done with technical duplicates.

We characterized the multinucleate cells in more depth by quantifying the precise number of nuclei per amoebae using direct counting. Interestingly, the distribution of the number of nuclei per cell was not random (Fig. 3). Bi- and tetra-nucleate amoebae were the most prevalent multinucleate types. Nuclei numbers equal to 3, 5, 6 and 7 were not detected in the tested conditions. The highest number of nuclei per *A.* *castellanii* observed was eight. Under agitated and adherent conditions, the few multinucleate amoebae were bi-and tetranucleated as well (Fig. 3).

To test if this generation of multinuclearity is specific to *A.* *castellanii* or if it represents a common mechanism shared by several Amoebozoa species, we also tested if the absence of cellular adhesion generated multinuclearity in the amoeba *Vermamoeba* *vermiformis*, which belongs to a different genus than *A.* *castellanii*. As *A.* *castellanii*, *V.* *vermiformis* has a biphasic life cycle, with trophozoites and cysts differentiated forms. It is commonly isolated worldwide from natural environments, such as freshwater surfaces but also from engineered systems such a tap water, swimming pools and hospital settings^14^. As shown in Fig. S1, agitated *V.* *vermiformis* amoebae under non-adherent conditions were also multinucleate. Quantification of the multinucleate cells after 24 h of agitation showed that 86.7% (± 5.7%) are multinucleate amoebae, while they represented a minor fraction of the population of adherent cells (5.6% ± 1.0%).

### A stretchy cytokinesis resolves multinuclearity

**Figure 4:**
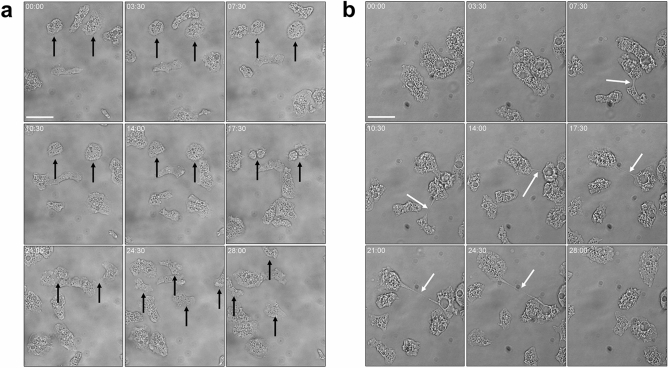
Time-lapse microscopy. Live amoebae previously non-agitated (**a**) or agitated (**b**) for 24 h were monitored for 28 min under light microscope using cytokinesis compatible conditions (without agitation). A set of 9 snapshots are shown for the two conditions. Black arrows show classical mitosis (**a**) while white arrows depict a “stretchy cytokinesis” for multinucleate amoebae (**b**). Scale bar: 50 µm.

During mitosis, an uninucleate *A.* *castellanii* amoeba rounds up, the nucleus replicates, becoming transiently non-visible under light microscopy, the cell divides at the middle, generating two uninucleate siblings of the same size, which separate from each other without cytosolic bridge (Fig. 4a). The situation is different for multinucleate *A.* *castellanii* cells. Once cellular adherence resumes (without agitation), multinucleate amoebae were able to quickly resolve their uninuclearity by a stretchy-like cytokinesis (Fig. 4b). In this specific case of cytokinesis characterizing multinucleate cells, the nuclei did not replicate and stayed visible throughout the division process, the amoebae did not round up and cytosolic bridges were formed (Figs. 4b and S2). Uninucleate sibling cells were budding from the mother cell, resolving the multinuclearity. Starting from a binucleate amoeba, each daughter cell contained one single nucleus, and they were not anucleate. The two nuclei localized at the opposite direction of the amoeba and the whole cell was teared apart, generating two linked cell bodies going in opposite direction, leading to the formation of a cytosolic bridge. Tensions appeared to accumulate in the bridge until it broke down, leading to the formation of two uninucleate daughter cells. Taken together with previous observations^15^, this suggests that a non-random separation process, associated with a regulatory mechanism and involving locomotion machinery is taking place in *Acanthamoeba* spp.

### Single cell quantification highlights a proliferation advantage

**Figure 5:**
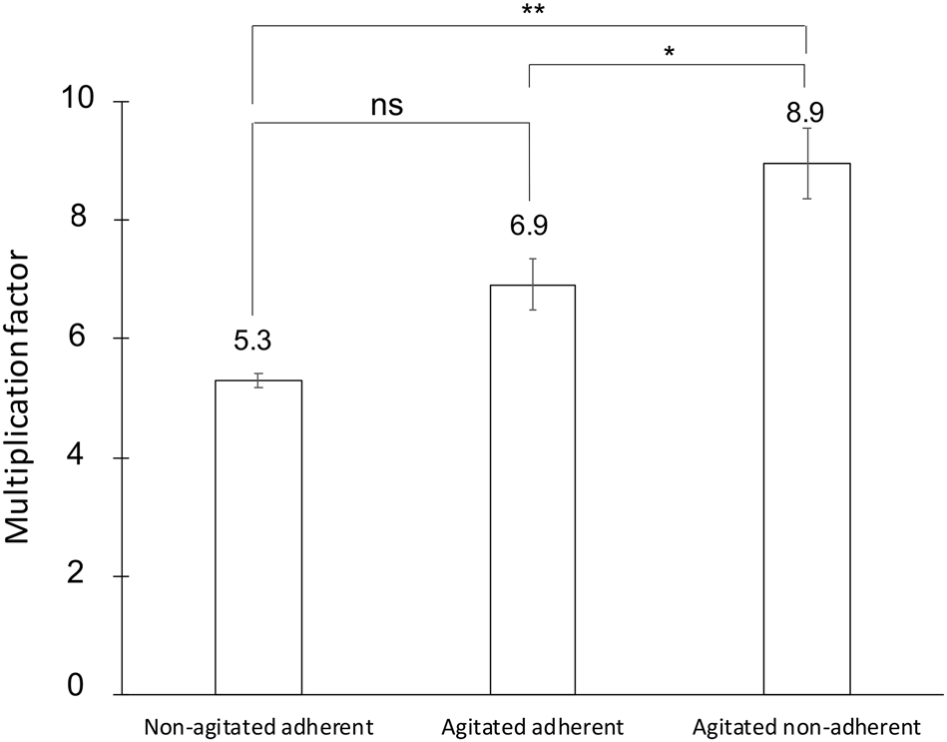
Growth advantage at the population level under axenic conditions. *A.* *castellanii* cells were allowed to adhere and replicate for 24 h without agitation. The multiplication factors represent the number of amoebae at 48 h divided by the number of amoebae at 24 h (see also Fig. 1 for workflow). Data are generated from three biological independent experiments, which were run using technical duplicates. Error bars ± SEM. (*) p < 0.05; (**) p < 0.01 and (ns) p > 0.05.

Next, we checked if multinucleate *A.* *castellanii* amoebae could have a population growth advantage compared to uninucleate cells by enumerating the number of single celled amoebae over time. We first measured the proliferation rate of the amoeba population starting with uni- or multinucleate cells (Fig. 1) in rich axenic conditions. The calculated multiplication factor represents the quantification of the sibling cells that originate from uni-or multinucleate amoebae when put back under cytokinesis permissive conditions (with adherence). It was calculated by dividing the number of amoebae at T48h by the number of cells initially present at T24h within the same well (Figs. 1, 5). While the uninucleate amoebae multiplied on average 5.30 (± 0.12 SEM) times in 24 h, the population of multinucleate amoebae showed an 8.95-fold (± 0.58 SEM) increase. Amoebae in control conditions (agitated but adherent), for which a lower number of multinucleate cells were present (Figs. 2, 3), gave an intermediate factor, with 6.92-fold (± 0.44 SEM) increase. Detached multinucleate amoebae, through a direct effect of the agitation conditions, could have participated in this moderate proliferation advantage. Taken together, these data show that under the tested conditions and using single cell enumeration over time, agitation-induced multinucleate amoebae gave rise to more progeny cells than non-agitated cells once adherence resumed.

**Figure 6:**
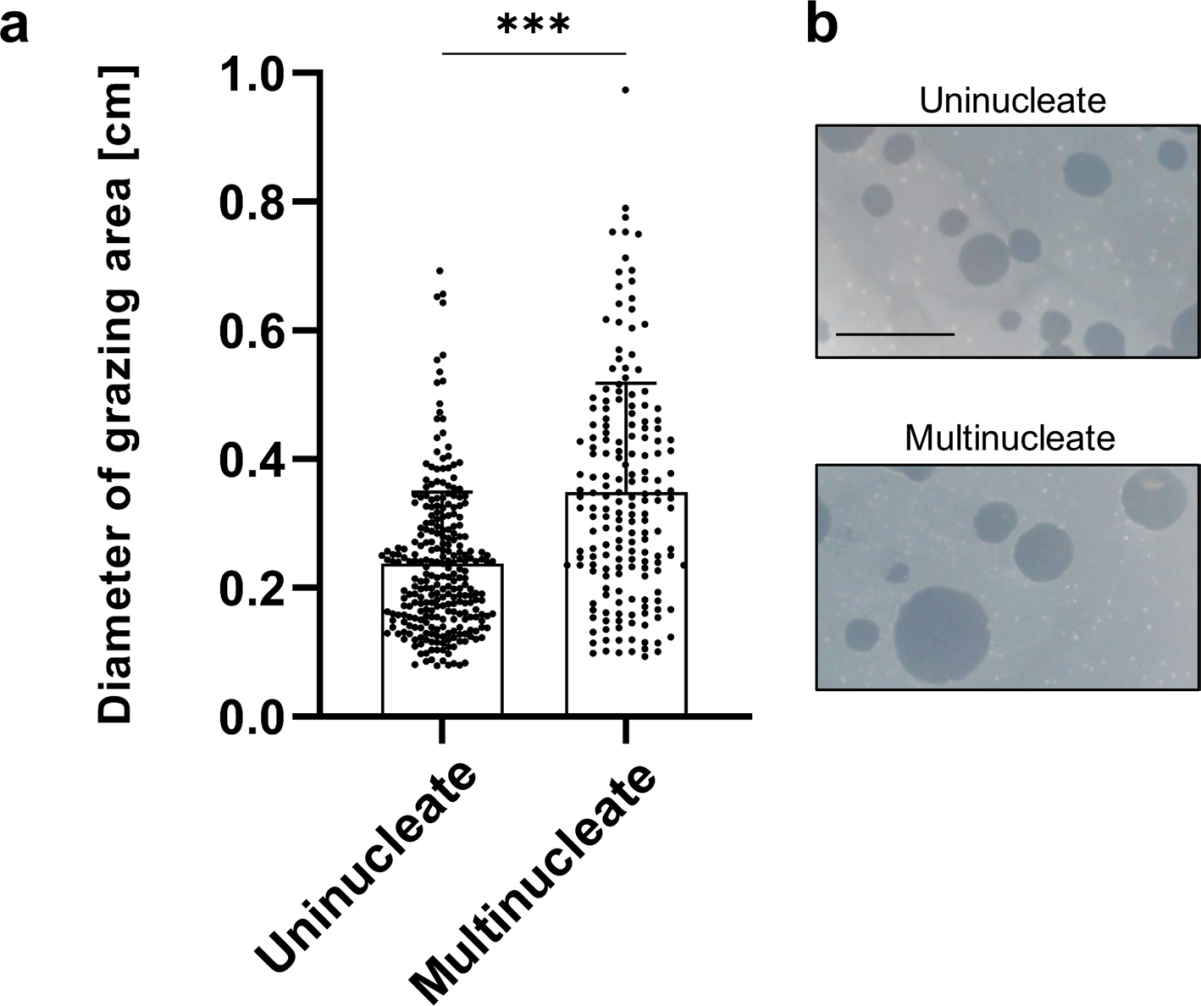
*A.* *castellanii* grazing on bacterial lawns. (**a**) Frequency distribution of grazing diameters of uninucleate and multinucleate *A.* *castellanii* amoebae. (**b**) Representative images of grazing zones obtained from solid medium coated with *E.* *coli* bacteria as food source, inoculated either with uninucleate (upper panel) or multinucleate amoebae (lower panel). Scale bar: 1 cm. Data were obtained from three biological independent experiments.

Amoebae are unicellular eukaryotic predators, feeding on bacteria, fungi and yeast cells, for instance^9^. We showed an increased population growth of multinucleate amoebae under rich and axenic laboratory culture conditions (Fig. 5). We next tested a potential proliferation advantage under more biologically relevant conditions by inoculating uni- or multinucleate amoebae on bacterial lawns. Relying on their phagocytic activity, *A.* *castellanii* amoebae can efficiently feed and proliferate on a layer of susceptible bacteria, generating the so called “grazing” zones that are cleared of bacteria^16^. The higher the diameter, the more efficient an initial single amoeba cell was able to feed on bacterial preys and proliferate actively, therefore increasing the population size. Measurements and subsequent quantification of the diameter of the grazing areas are given in Fig. 6. The grazing areas started from multinucleate amoebae displayed a higher diameter compared to uninucleate cell origin (Fig. 6). According to our previous observations under axenic conditions, these observations show that multinucleate amoebae were more prone to colonize a growth-permissive substrate under non-axenic conditions as well.

## Discussion

We showed that agitating conditions promoted multinuclearity in *A.* *castellanii* and *V.* *vermiformis* by generating a cytokinesis delay. Interestingly, multinuclearity induced by agitation has been shown for other aquatic protists as well, such as *Naegleria* *gruberi*^4^ and *Hartmannella* *rhysodes*^5^. Tetranucleated cysts are part of the infection cycle of *E.* *histolytica*^1^. Based on the data presented in our study, we can suggest that this specific multinucleate stage in *E.* *histolytica* could provide a growth advantage during infection that allows this pathogenic amoeba to efficiently produce numerous descendants when colonizing a new host*.* This hypothesis remains to be tested. In this light, the involvement of multinucleate cells in *A.* *castellanii* pathogenicity deserves to be investigated, and direct single-cell level analyses on human samples, instead of classical amoeba isolation protocols^17^, could render these observations possible.

The strain used in our study is the *Acanthamoeba* *castellanii* (Douglas) ATCC30010 (strain Designations: Neff, Depositor RJ Neff, biosafety level 1). This *A.* *castellanii* isolate has kept its cell cycle related ability to encyst, giving rise to non-adherent mature cysts under starvation conditions^18,19^. In addition, this isolate retains its capacity to efficiently feed on bacterial preys. Taken together, these observations show that the validated amoebal isolate used in our study is not axenically adapted, at least to the extents regarding its cell cycle and phagocytosis processes.

At least two different, yet non-exclusive mechanisms can generate multinuclearity (Fig. S3). Firstly, uninucleate amoebae could fuse together leading to the formation of a multinucleate cell, therefore forming a syncytium. This hypothesis is interesting knowing that *A.* *castellanii* are asexual organisms replicating through mitosis^15^, while possessing meiotic genes^20–22^. Reuniting multiple nuclei, originating from different cells, in a single amoeba could promote genetic material exchange and create diversity, essential for evolution. In this light, the amoeba *Cochliopodium* spp. was previously reported to undergo cellular followed by nuclear fusions^23^. In case of cellular fusions of uni- and multinucleate amoebae occurring randomly in *A.* *castellanii*, we would expect another distribution of the number of nuclei per amoeba including even and odd numbers, which is not what we report in our study (Figs. 3 and S3). A potential mechanism that regulates particular *A.* *castellanii* cell types to fuse together, such as the mating types designated MATa and MATα in yeast^24^, cannot be excluded at this stage. Also, if binucleated cells are more prone to fuse with binucleated cells, this would generate tetranucleated cells. Using live cell imaging and time lapse microscopy, we never observed such fusion events. However, at this stage, we cannot exclude that the frequency of cellular fusion is too low to be detected in our conditions or that the tested conditions in our laboratory did not promote fusion. A second hypothesis proposes that multinuclearity originates from endoreplication, consisting of synchronous nuclei replication without cytokinesis, successively generating from an uninucleate cell (1n) polyploid cells such as 2n, 4n, 8n, … (Fig. S3). The fact that under non-adherent conditions bi- and tetra- nucleated amoebae represent the vast majority of the population (and to a minor extent 8 nuclei) provides support for this second hypothesis (Figs. 2, 3). In our study, no case of uneven nuclei per amoeba were observed (except for single nucleated amoebae). The endoreplication hypothesis is supported by the fact that amoebae must be adherent to their substrate to divide, because the locomotion system is important to tear apart the daughter cells^5^. Agitation would impair such adhesion, and multinuclearity would be a consequence of amoebae trying to divide but ultimately failing at the cytokinesis stage, resulting in 2^n^ number of nuclei. This endoreplication hypothesis does not exclude the fusion hypothesis and both phenomena could occur concomitantly at different rates, depending on the tested conditions.

**Figure 7:**
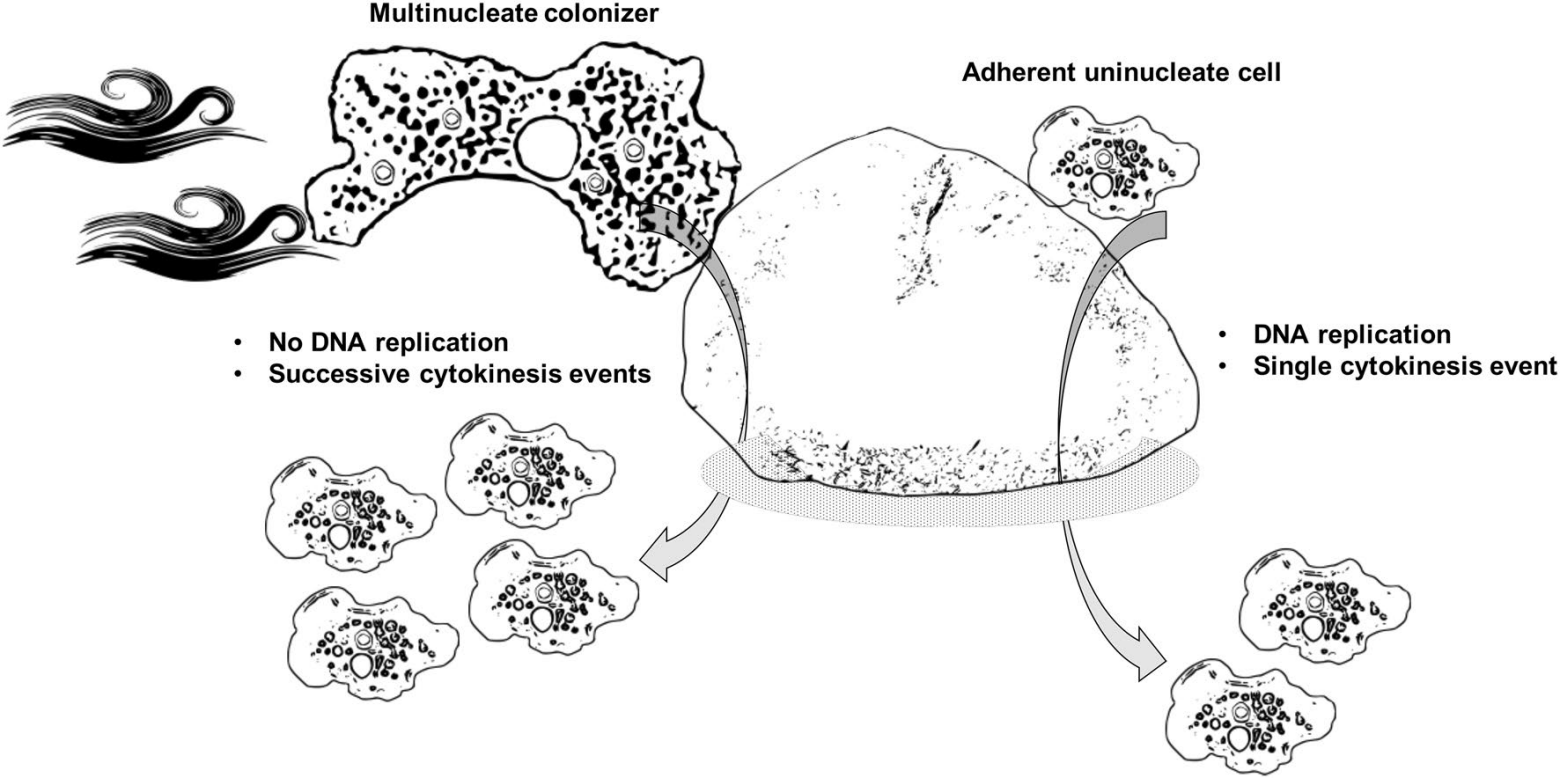
Model of multinuclearity associated to a potential environmental fitness. Schematic representation of a model starting from two single-celled amoebae with different origins competing for the same resources present in a new favourable niche. One is a multinucleate amoeba with four nuclei (left side) and the other one is an adherent uninucleate cell, all else being equal. Both amoebae arrive and adhere concomitantly on a surface to-be-colonized. After successive cytokinesis events only, without the need of DNA replication that occurred before, the multinucleate amoeba gives rise to four sibling cells. However, the uninucleate amoeba has to replicate its genetic content prior to divide through a mitotic process, generating two progeny cells only. As the progeny cells will enter mitosis and proliferate, the population that originates from a multinucleate mother cell will be constantly more numerous.

Producing a higher number of clonal progenies for the multinucleate cells (Fig. 5) and a better substratum colonization (Fig. 6) is equivalent to a greater individual reproductive success, which is one of the aspects of a fitness advantage^25,26^. Upon cellular attachment to a new solid surface-to-be-colonized and based on cytokinesis events only (without the need to replicate its genome at that moment), multinucleate amoebae can generate sibling cells faster than uninucleate amoebae, all else being equal (Figs. 3, 5, 7). The observed generation advantage takes place specifically upon the process of new substrate colonization. Indeed, multinucleate amoebae had to replicate their DNA content before, as for uninucleate ones, and if we take this into account, it might be possible that no fitness advantage could be highlighted. Nevertheless, when focusing on the colonization timing, when two amoeba cells arrive together (uni-versus multinucleate) and compete for resources, multinucleate cells do have a colonization advantage as they do not need to replicate their DNA content, resulting in a faster division accompanied by an increased population growth (Fig. 7). In addition, the observation that multinucleates are giant cells might help amoebae to sediment and adhere to their new substrate faster than uninucleate ones. This observed advantage relies on the quantification of the number of single celled amoebae and might differ at the level of biomass or total nuclei number.

Concerning the non-adherent conditions, as *A.* *castellanii* amoebae are frequently found in aquatic environments, it is possible that in their natural niche, *A.* *castellanii* cells regularly detach from a biotic or abiotic surface. For example, an early step during encystation is cell detachment. Before this differentiation process being irreversible^9,11^, detached amoebae could have the time to be transported in the water column to colonize a new environment. In addition, any flux perturbations, water currents or waves might detach amoebae from their substrate and suspend them in water, in a non-adherent state. This process might help amoebae to colonize new environmental niches that are difficult to reach using an adherent state. This cellular detachment is seen in our control conditions when adherent amoebae are agitated, which leads to a small percentage of detached multinucleate amoebae even if the majority of the population remain adherent and uninucleate (Fig. 3). Detached amoebae would therefore get disseminated by water currents, replicating their DNA content without cytokinesis, preparing themselves to efficiently colonize a new habitat. To test this hypothesis, such colonizers could be collected directly from natural environmental water columns. However, the current isolation methods do not allow any direct and high-throughput single cell based-observations of collected amoebae, together with their precise identification, as they are rather based on isolation of amoebae through enrichment on bacterial lawns^17^. As shown in our study, even if any multinucleate amoebae are collected and inoculated on solid media from natural environment, the multinuclearity could be lost after a few stretchy cytokineses.

Excessive cell growth leads to cytoplasm dilution that impairs cellular processes such as cell signalling and translation^27^. There is an optimal DNA/cytoplasm ratio, and values outside the tolerance zone impair cellular fitness as RNA and protein synthesis become limiting, leading to cellular senescence^27^. Giant multinucleate cells could therefore face cytoplasm dilution. Hence, as a physiological response, amoebae may increase the number of nuclei to counteract the cytoplasm dilution phenomenon.

## Conclusion

In conclusion, we show that agitation delays cytokinesis process, which induces multinuclearity in *A.* *castellanii* Neff strain. The number of nuclei per multinucleate cell is not random (bi-tetra and octa-nucleated amoebae are observed) and each daughter cell receives a nucleus, strongly suggesting that a non-random cytokinesis process is taking place. When agitation stops, amoebae solve their multinuclearity by generating cytosolic bridges that give rise to multiple uninucleate daughter cells. Hence, multinucleate amoebae generate a higher progeny population compared to uninucleate mother cells once cellular adherence resumes and all else being equal. This advantage is observed upon quantification of the number of individual cells over time. We propose that multinuclearity should not be considered as an artefact in amoebae, but rather as a physiological adaptation to environmental conditions, potentially generating better colonizer cells upon absence of adhesion. A direct consequence in amoebae resulting in an advantage that can contribute to a faster colonisation during niche competition.

## Materials and methods

### Stains and media

The amoebal strain *Acanthamoeba* *castellanii* (Douglas) ATCC30010 was cultivated in peptone yeast glucose medium (PYG) (ATCC medium 712) in T75 cell culture flasks (ThermoFisher, MA, US) with 10 ml of PYG at 25 °C (± 1 °C). To avoid any axenic adaptation at best, the amoebal cultures were regularly allowed to encyst prior to dilution in rich medium for the next cellular amplification. Also, the amoebal culture was use for 30 passages at the maximum, then discarded and a new amoebal culture from a frozen stock solution was launched. *Vermamoeba* *vermiformis* ATCC50237 were grown in PYNFH medium (bacto-peptone 1%, yeast extract 1%, yeast nucleic acid (Ribonucleic Acid, Type VI from Torula Yeast, Sigma) 0.1%, folic acid 33 μmol L^−1^, hemin 1.5 μmol L^−1^, Na2HPO4 3.6 mmol L^−1^, KH2PO4 26 mmol L^−1^, foetal bovine serum heat-inactivated 10%, pH = 6.5) at 30 °C. *Escherichia* *coli* strain S17-1λpir, used as a food source for amoeba grazing experiments, were grown in LB (Invitrogen, CA, US) for 16 h at 37 °C under agitation (175 rpm). When amoeba cultures reached saturation, meaning 100% confluence that corresponds to a calculated number of 2 * 10^6^ cells/ml (using a KOVA chamber), amoebae were scraped, gently homogenized and 1 ml was transferred to a new T75 flask with 9 ml PYG for 24 h. This corresponds to the “T0” of all experiments (Fig. 1). This step ensures the synchronisation of the population in the trophozoite form, with the same physiological state and growing phase, and that the same number of *A.* *castellanii* cells is engaged prior to the experiment.

For the “non-agitated adherent” control conditions, *A.* *castellanii* cells were inoculated in a 6 wells plate with 2 ml of PYG and incubated at 25 ± 1 °C without agitation. For the “agitated non-adherent” conditions, *A.* *castellanii* amoebae were inoculated in 6 wells plate with 2 ml of PYG and incubated at 25 ± 1 °C for 24 h under agitation (250 rpm). For the quantification of the number of nuclei per amoebae, agitated *A.* *castellanii* were allowed to adhere for one hour prior to direct visualization using light microscope. Observations consist of taking at least 8 random micrographs per well, throughout the entire well, to avoid any edge effects. For the “agitated adherent” conditions, *A.* *castellanii* were scrapped from the initial stock culture and put in a 6 wells plate with 2 ml of PYG without agitation for 3 h. This step allows the adherence of the amoeba cells. After that, the plate was put under agitation (250 rpm) for 24 h at 25 ± 1 °C.

Regarding *V.* *vermiformis*, the “non-agitated adherent” control cells were inoculated in a 6-well plate with 2 ml of PYNFH and incubated at 30 °C without agitation. For the “agitated non-adherent” conditions, *V.* *vermiformis* were inoculated in 6-well plates and incubated at 30 °C for 24 and 48 h under agitation (250 rpm). For the quantification of the number of nuclei per amoeba, cells were scraped and centrifuged at 1,000 g for 10 min. Cells were suspended and fixed using a Page’s Amoeba Saline solution (4 mM MgSO4, 0.4 M CaCl2, 0.1% sodium citrate dehydrate, 2.5 mM NaH2PO3, 2.5 mM K2HPO3, pH 6.5) containing 2% of paraformaldehyde for 10 min. After a new centrifugation, cells were suspended in glycerol/PBS solution (CitiFluor™ AF1) containing 0.5 µg/mL µM of DAPI (Sigma-Aldrich). Nuclei were observed under inverted epifluorescence microscope (Olympus IX73).

### Scanning electron microscopy (SEM)

Amoebae observed under SEM were incubated under agitation in 50 ml tubes (Merck, Darmstadt, Germany) with 4,7 ml of PYG and 300 µl of scrapped amoebae from a pre-culture at stationary phase (which corresponds to a calculated concentration of 2 * 10^6^ amoebae/ml). Amoebae were put in a temperature-controlled agitator (350 rpm) at 25 °C (± 2 °C) for 24 h. Agitated amoebae were then put at rest (without agitation) prior to be scrapped, put on cover slips and dehydrated through successive ethanol baths of increasing concentrations as follows: samples were incubated in 30%, 50%, 70%, 85% and 100 ethanol two times each for 5 min and 10 min, except for the last step, which was 3 times 10 min incubation. Then, ethanol was replaced with hexamethyldisilazane (two times 15 min) that evaporated overnight under a chemical hood. Samples are covered with 25 nm of Gold using a Quorum Q150T/ES sputter coater and then analysed under a JEOL 6,010 LV SEM at 15 kV.

### Amoeba grazing

Grazing assays were done on solid medium using 25 ml of LB agar diluted 5 times (Merck, Darmstadt, Germany) per petri dish. Stationary growing phase *E.* *coli* were diluted with liquid LB to reach an OD of 0.5 (at 600 nm). A calculated number of 200 *A.* *castellanii* cells were mixed together with the bacteria in a final volume of 200 µl. The 200 µl mix of bacteria and amoebae was then plated using a plastic rake on LB agar plates and put under the hood to dry for about 15 min. Finally, the plates were incubated at 25 °C for 6 days. Grazing diameters were measured using imageJ software^28^.

### Statistical analyses

Data used for the enumeration of uni-versus multinucleate cells were generated from three independent biological replicates. At each time point, at least 8 micrographs were randomly taken. Amoebae were counted using imageJ software^28^. The number “n” of amoebae counted for the counting of uni- and multinucleate cells was: n_(non-agitated and adherent)_ = 1511, n_(agitated adherent)_ = 181, n_(agitated non adherent)_ = 365. When the number of nuclei per amoeba was unclear (i.e. out of the focal plan or not entirely visible in the field of view), they were not counted. We estimated that the fraction of blurry or incomplete amoebae represented maximum 15% of the total number of the counted amoebae. The population growth and the number of nuclei were analysed using ANOVA and Scheffé’s methods. Data used in Figure S1 were obtained from three independent biological replicates. For each condition, at least 100 cells were randomly counted. All histograms are an average of three biologically independent experiments and error bars represent the standard error of the mean (SEM). Concerning the grazing area generated from uni-versus multinucleate amoebae, an unpaired t test, two-tailed, with Welch’s correction has been executed with GraphPad Prism Software (San Diego, CA, USA) to compare the grazing distribution that is highly significant (p value < 0.0001).

## Supplementary information

Supplementary file1 (PDF 2704 kb)
