# Supplementary material for: Delayed cytokinesis generates multinuclearity and potential advantages in the amoeba Acanthamoebacastellanii Neff strain
Source: Sci Rep. 2020 Jul 21;10:12109. doi: 10.1038/s41598-020-68694-9 (PMC7374626; doi:10.1038/s41598-020-68694-9)
Supplement: Supplementary file 1 — Supplementary file1 (PDF 2704 kb) [file 41598_2020_68694_MOESM1_ESM.pdf]

Delayed cytokinesis generates multinuclearity and potential advantages in the amoeba *Acanthamoeba castellanii* Neff strain

Théo Quinet[1], Ascel Samba-Louaka[2], Yann Héchard[2], Karine Van Doninck[1] & Charles Van der Henst[1,3,4,5*]

Affiliations

[1] Laboratory of Evolutionary Genetics and Ecology, URBE, University of Namur, Belgium.

[2] Laboratoire Ecologie et Biologie des Interactions, Equipe Microbiologie de l'Eau, Université de Poitiers, UMR CNRS 7267, F-86073 Poitiers, France.

[3] Research Unit in the Biology of Microorganisms (URBM), NARILIS, University of Namur (UNamur), Belgium.

[4] Microbial Resistance and Drug Discovery, VIB-VUB Center for Structural Biology, VIB, Flanders Institute for Biotechnology, Brussels, Belgium.

[5] Structural Biology Brussels, Vrije Universiteit Brussel (VUB), Brussels, Belgium.

***Corresponding author:** charles.vanderhenst@vub.vib.be

Dr. Charles Van der Henst, VIB-VUB Group leader, Microbial Resistance and Drug Discovery, Flanders Institute for Biotechnology (VIB), Vrije Universiteit Brussel (VUB), Center for Structural Biology (CSB), Pleinlaan 2, Building E-3, 1050 Brussels (BE), +32 (0) 2 629 19 40

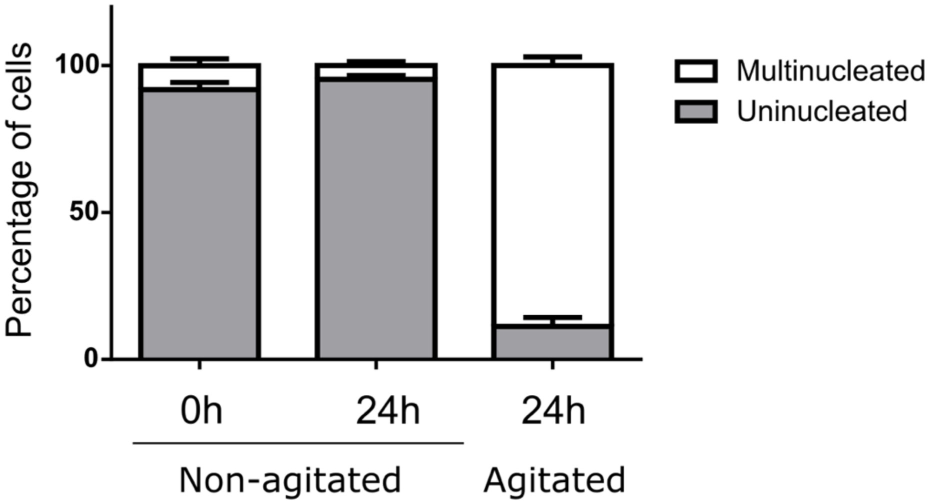

Figure S1: Multinuclearity induced in *V. vermiformis*. Percentage of multinucleated *V. vermiformis* under agitation (Agitated) or not (Non-agitated) for 0h and 24h. Results are averages of three independent experiments, and error bars represent the standard error of the mean (±SEM). Statistical analysis was performed using the Unpaired t-test (*** $p < 0.0001$).

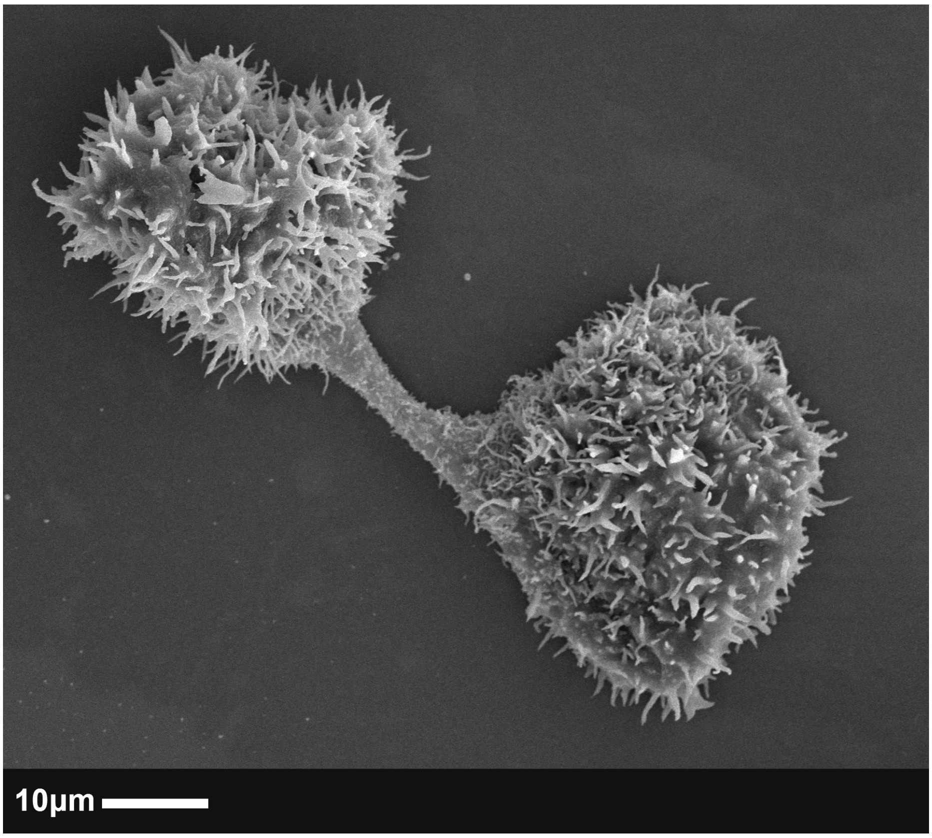

Figure S2. Cytosolic bridge of *A. castellanii*. Scanning electron microscopy of a multinucleate amoeba cell undergoing cytokinesis. A cytosolic bridge characteristic of multinucleate dividing cells is visible in between the two cell bodies. Scale bar: 10 µm.

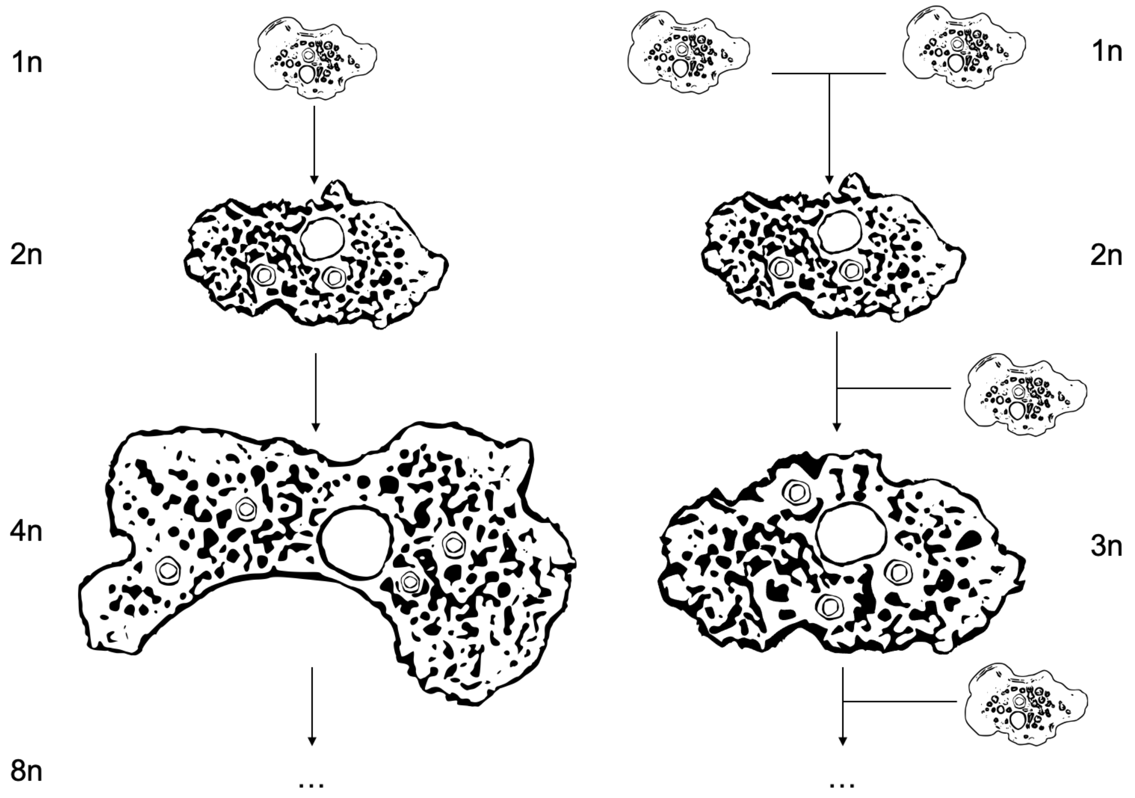

Figure S3. Potential origins of cellular multinuclearity. Multinucleate cells can be generated by endoreplication (left panel), for which the DNA content replicates synchronously in the absence of cytokinesis or by successive fusion events (right panel).
